# Lumbar trans-spinal direct current stimulation: A modeling-experimental approach to dorsal root ganglia stimulation

**DOI:** 10.3389/fnins.2022.1041932

**Published:** 2022-12-08

**Authors:** Mariana Pereira, Sofia Rita Fernandes, Pedro Cavaleiro Miranda, Mamede de Carvalho

**Affiliations:** ^1^Instituto de Medicina Molecular, Faculdade de Medicina, Universidade de Lisboa, Lisboa, Portugal; ^2^Instituto de Biofísica e Engenharia Biomédica, Faculdade de Ciências, Universidade de Lisboa, Lisboa, Portugal; ^3^Departamento de Neurociências e Saúde Mental, Hospital de Santa Maria - Centro Hospitalar Lisboa Norte, Lisboa, Portugal

**Keywords:** tsDCS, spinal cord, dorsal ganglia, neurophysiology, computational modeling, electric field

## Abstract

**Introduction:**

The excitability of spinal motor neurons (MN) can be altered through subthreshold currents, such as transcutaneous spinal direct-current stimulation (tsDCS). Current evidence shows that tsDCS can interfere with ascending somatosensory pathways and lower motor neurons’ (LMN) excitability, which points to its therapeutic potential for repairing altered spinal responses. We aim to define the best tsDCS montage for maximizing the electric field (E-field) in the lumbar spinal cord (L-SC) by computer modeling; and to apply this montage to measure the effect on LMN excitability and somatosensory evoked potentials (SSEP).

**Methods:**

A human volume conductor model was obtained from an available database. The E-field distribution was calculated considering three different electrode settings aiming at maximizing the field at L-SC and right dorsal root ganglia (DRG). The best electrode setting was then selected and applied in a blind crossover pseudo-randomized study including 14 subjects. tsDCS was delivered for 15 min (cathodal vs. sham) over L2 vertebra level (4 mA, 144 mC/cm^2^), and its effect on F-waves, H-reflex (including homosynaptic depression, HD) and SSEPs was investigated in the lower limbs.

**Results:**

All simulated montages showed higher current density and E-field magnitudes between the electrodes (>0.15 V/m), with a major longitudinal component and with rostral-caudal direction. The induced E-field involved the sensory ganglia and was maximum in the right T8-left L2 montage, which was the one selected for the experimental protocol. We disclosed a statistically significant increase of the H-reflex amplitude at 0.1 Hz, after cathodal tsDCS (c-tsDCS) on both sides. No other significant change was observed.

**Discussion:**

Our results can suggest the c-tsDCS applied to the L-SC and DRG can modulate synaptic efficiency increasing lower motor neurons response to Ia fibers excitation. The possible implications of our findings for treating clinical conditions will be addressed in future studies.

## Introduction

The spinal cord (SC) is a complex structure with two main functions: communication between the brain and peripheral structures; integration of reflexes essential for many functions, such as posture and movement. Dysfunction of specific spinal circuits is identified to be involved in many neuropathological conditions such as spasticity observed in patients with upper motor neuron (UMN) diseases (Lance, 1990). Pathologically-derived changes in descending inhibitory and excitatory pathways can influence the spinal reflexes (Lundberg, 1979), by interfering with the interplay between interneurons (IN) in the SC and decreasing the post-synaptic inhibition, thus impacting lower motor neuron (LMN) excitability (Hofstoetter et al., 2014).

In this sense, there has been a growing interest in modulating the excitability of the motor neurons (MNs) directly or indirectly, by changing its excitatory (EPSP) or inhibitory postsynaptic potential (IPSP), or by influencing the descending neuromodulatory pathways. Recently, the successful stimulation of the dorsal roots ganglia (DRG) to treat spasticity has been reported by using radiofrequency (Chang and Cho, 2017) and invasive DRG stimulation (Soloukey et al., 2020). Also, low-frequency (1 Hz) stimulation applied to DRGs was showed to be the most optimal for pain therapy in an animal model of diabetic polyneuropathy when compared to mid- and high-frequency (Koetsier et al., 2020). Possible underlying mechanisms include long-term synaptic depression and decrease of the afferent input (Chang and Cho, 2017).

Transcutaneous spinal direct current stimulation (tsDCS) is a non-invasive subthreshold neuromodulatory technique that induces an electric field (E-field) in the SC, with the purpose of inhibiting or facilitating neuronal responses by transiently changing the resting membrane potential of spinal neurons. Just as in brain stimulation, the relative orientation of the spinal neurons relative to the E-field induced by tsDCS will determine the effective modulation. Since 2014, several computational studies predicting the current and E-field induced in the SC by tsDCS in realistic human models were published (Parazzini et al., 2014; Fernandes et al., 2015, 2019; Kuck et al., 2017; Pereira et al., 2018). These studies are important to clarify the most effective experimental protocol for specific clinical purposes (Fernandes et al., 2019). Understanding the functional complexity of the motor spinal circuits and the orientation of the longitudinal and transverse fibers in the SC is determinant to understand the possible therapeutic effects of tsDCS as a non-invasive repair strategy (Creutzfeldt et al., 1962; Ahmed, 2011).

The aim of our study was to guide the application of tsDCS targeting the lumbar SC segments and corresponding DRG. Our hypothesis is that DC currents may also be able to modulate DRG-related responses, as previously observed in low-frequency stimulation studies reported above. We started by first modeling the E-field distribution in three electrode montages, and then applying the montage that maximizes the E-field at target (DRG locations) in an exploratory clinical study to ascertain tsDCS effects on the excitability of the spinal MNs.

## Materials and methods

This study combines modeling (I) and experimental components (II), with methodology described in the two following sections.

### Computational study methodology

#### Human model and electrode design

A realistic human model was designed and adapted from the 34 years-old Duke from the Virtual Population Family (Christ et al., 2010), considering 13 tissues: skin, fat (including subcutaneous adipose tissue, SAT), muscle, bone, heart, lungs, viscera (stomach, liver, pancreas, small intestine, and large intestine), dura mater, vertebrae, intervertebral disks, cerebrospinal fluid (CSF), brainstem, and SC. Design procedures and mesh operations are described in our previous work (Fernandes et al., 2018, 2019). Briefly, tissue assembly and tetrahedral volume mesh creation were performed with the 3-MATIC module from MIMICS (v16),^1^ with the full model truncated at the level of the thighs and above the elbows, to shorten computational time. The spinal gray matter (spinal-GM) was artificially designed considering standard knowledge on spinal cord anatomy (Standring et al., 2008) and measurements from the Visible Human Data Set (VHD data) of the National Library of Medicine (NLM) and the Visible Human Project^®^.^2^

**FIGURE 1:**
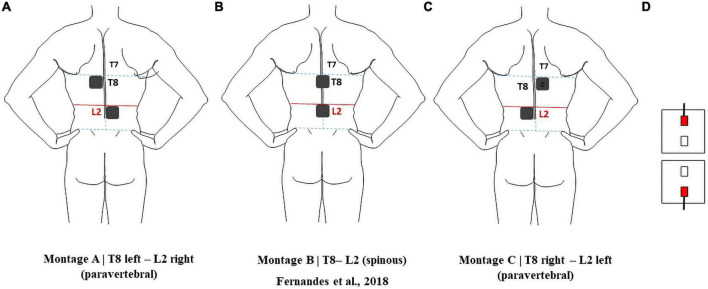
Electrode montages considered in this report: **(A)** pvT8 left-pvL2 right; **(B)** T8-L2; **(C)** pvT8 right-pvL2 left. **(D)** Relative position of the active connectors in all montages.

Electrodes were modeled according to the electrodes available in our lab (Fiab Spa, Vicchio, Italy),^3^ with a gel layer and rubber pad with a half-cylinder volume bearing a cylindrical metallic connector inside, 10 mm wide and 1 mm in diameter. The gel layer and the rubber pad base were considered as rectangular prisms 5 cm × 5 cm × 0.25 cm and 4.9 cm × 4.9 cm × 0.1 cm, respectively. The electrodes were placed between L1 and L2 spinous processes or 2.5 cm right or left paravertebral and over or 2.5 cm right or left paravertebral to the T8 spinous processes, respectively (Figure 1). Electrode placement was chosen to comprise the main target of interest in this study, i.e., the lumbosacral intumescence, region from where the sciatic nerve roots emerge. Paravertebral placement was chosen to avoid the low conductive bony spinous processes of vertebrae in the current’s path, and to observe if paravertebral placement can induce a larger right-left E-field component (E_*rl*_) comparatively to the T8-L2 spinous montage. The electrodes were then assembled to the human model in 3-MATIC, resulting in 1.3 × 10^7^ tetrahedral volume elements for each of the three models with electrodes. Further refinements of the surface meshes to address convergence of variables were not possible to perform in COMSOL due to the complex anatomical features of the model. Manual refinements were performed in 3-matic, to get the most of the model. A successful volume mesh was only obtained for maximum triangle length for spinal white matter (spinal-WM) and spinal dura surfaces of 1 mm combined with 4 mm for the remaining tissues. No further refinements resulted in a successful mesh.

#### Properties of tissue and electrode materials

Isotropic electric conductivity values of tissues were compiled from a literature review on DC current tissue properties (Table 1). Anisotropy of muscle and spinal-WM conductivity was modeled by tensors using information about known spatial orientation and considering the volume constraint from Wolters (2003), as described in Fernandes et al. (2018). The conductivity matrix was calculated in each mesh node using a custom-made Matlab script (v2015b).^4^ Each node conductivity matrix components were interpolated in COMSOL Multiphysics (4.3b)^5^ to obtain the anisotropic conductivity tensor for each volume element. The gel was assigned a conductivity of 4 S/m, according to Minhas et al. (2010) and the rubber pad conductivity was measured using the four-point probe method and estimated to be 44 ± 1 S/m (Pereira et al., 2018).

**TABLE 1:** Electric conductivities of tissues considered in the human model.

Tissue	σ (S/m)	References
Skin	0.435	Geddes and Baker, 1967
Fat	0.040	Haueisen et al., 1997
Muscle (isotropic)	0.355 (av)	Rush et al., 1963
Lungs	0.046 (av)	Rush et al., 1963
Heart	0.535 (av)	Surowiec et al., 1987; Haueisen et al., 1997
Viscera (liver, pancreas, stomach, small and large intestine, and air)	0.123 (av)	Surowiec et al., 1987; Haueisen et al., 1997
Vertebrae/Bone	0.006	Haueisen et al., 1997
Intervertebral disks	0.200	Haueisen et al., 1997
Dura mater	0.030	Struijk et al., 1993
CSF	1.790	Baumann et al., 1997
Brainstem/spinal roots	0.154	Haueisen et al., 1997
Spinal-WM (isotropic)	0.143	Haueisen et al., 1997
Spinal-GM	0.333	Haueisen et al., 1997

#### Electric field calculations

Electric field calculations were performed with COMSOL Multiphysics using the finite element (FE) method. The current intensity in the electrodes was set to 4 mA, according to the value to be applied in the experimental setting. The boundary conditions were applied according to Miranda et al. (2013), considering the cylindrical metallic connector surface as isopotential. The target electrode was considered as cathode and the return electrode as anode. Reversing polarity would invert the direction of the E-field but would not affect its magnitude or the magnitude of its components (Ruffini et al., 2013).

Modeling studies on tDCS reported average E-field values greater than 0.15 V/m in the motor cortex when reproducing clinical settings with observed neuromodulatory effects, thus, we will consider that tsDCS neuromodulatory effects may possibly occur when the E-field exceeds this value in the SC (Nitsche and Paulus, 2000; Miranda et al., 2013).

### Clinical study methodology

#### Subjects

We included 14 healthy right-handed volunteers recruited from university students and staff, with a mean age of 30 years (ranging from 23 to 63, male-female ratio of 1:3). Subjects with neurologic, metabolic, and psychiatric diseases, previous spinal surgery or taking drugs that could influence neuronal excitability were excluded.

The protocol was approved by the local ethics board (reference 405/2019) and all subjects provided a written informed consent.

#### Materials

Skin surface was cleaned with abrasive gel before applying simulating and recording electrodes. For recording, pre-gelled, disposable, surface electrodes (recording area 4.9 cm^2^) were used for motor responses, while conventional gold cups electrodes (Genuine Grass^®^ Reusable Cup EEG Electrodes) were used for somatosensory evoked potentials recordings (PESS). The same gel (Signa Gel^®^, Parker, USA, electrode gel) was used on gold cups and tsDCS electrodes. All electrodes were fixed with adhesive tape. Motor and sensory responses were recorded with impedance values below 10 kΩ (Rossini et al., 2015). Abnormal impedance values or noise artifacts were corrected by cleaning the skin surface and/or replacing the recording electrodes. All neurophysiological parameters were recorded with a conventional electromyography (EMG) equipment (Keypoint^©^, Natus Inc.).

#### Transcutaneous spinal direct current stimulation experimental protocol

Transcutaneous spinal direct current stimulation was applied by a battery powered stimulator (Soterix Medical^©^ tsDCS, USA) connected to a pair of square electrodes with 1 mm thickness and area 25 cm^2^ (5 cm × 5 cm). Impedance was continuously checked during current delivery.

Active stimulation (cathodal) was applied using a direct current of 4 mA during 900 s, resulting in a total current and charge density delivery of 16 mA/cm^2^ and 144 mC/cm^2^, respectively. These values are well below the safety threshold of 250 A/m^2^ for cutaneous and nervous tissues lesions(Liebetanz et al., 2009; Cogiamanian et al., 2011). Cathodal tsDCS (c-tsDCS) polarity refers to the electrode placed over the lumbar region. Placebo (sham) tsDCS was delivered during 900 s with intensity 0 mA.

The selected montage derived from modeling part I was applied in each subject (see section “Results”).

After recording baseline values (T0), the subjects underwent tsDCS stimulation (sham or cathodal), for 15 min. Subjects were randomized to sham or cathodal stimulation on different days (time interval >5 days) to avoid cross-over effects (Nitsche et al., 2003). All subjects were blinded to the tsDCS polarity, but not the researcher.

**FIGURE 2:**
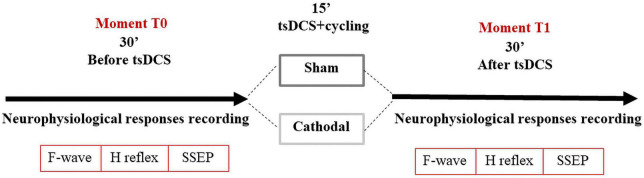
Study design.

Considering that legs immobility change some neurophysiological parameters, in particular F-waves latencies in lower limbs (Pereira et al., 2018, 2022), all subjects were asked to cycle during the 15-min of tsDCS (placebo or active stimulation), at a constant velocity (between 1.6 and 1.8 m/s) using a commercial cycle ergometer (Deluxe Pedal Exerciser, Chattanooga Group, USA, Model#18010), as described elsewhere (Pereira et al., 2022). After stimulation with exercise all 14 subjects were re-evaluated (T1). In total each session lasted 80–100 min (Figure 2).

#### Neurophysiological recordings

Limbs were kept warm (skin temperature higher than 31°C) and room temperature was maintained at 21–23°C, as suggested in the literature (Kimura, 1989). All subjects remained seated in a comfortable and slightly inclined chair. Lower limbs were positioned with 120° hip flexion, 160° knee flexion and 110° plantar flexion of the ankle (Pierrot-Deseilligny and Burke, 2012). The tested muscle was fully relaxed and in neutral position. Muscle relaxation was controlled by the device loudspeaker.

M and F-waves were recorded from both abductor hallucis muscles (AH) using a belly-tendon montage. For F-wave studies, 30 supramaximal percutaneous stimuli were applied at the ankle (variable interstimulus interval of 1–3 s). The ground electrode was placed between the stimulating and recording electrodes. F-waves were accepted if of amplitude >20 μV, with a filter bandwidth of 20 Hz–10 kHz (Pereira et al., 2018, 2022).

The H reflex was investigated in both legs, and obtained by delivering 1 ms rectangular stimuli in the popliteal fossa and recording over the soleus muscle (at the midpoint of the line joining the anterior tibial eminence to the internal malleolus), with the reference electrode over the Achilles tendon (Kimura, 1989). The time base and gain were set to 10 ms and 100–1,000 μV/division, respectively. The filter setting was 20 Hz–10 kHz. Stimuli frequency was less than 0.5 Hz to avoid post-activation depression (Pierrot-Deseilligny and Burke, 2012) and stimulus duration was 1 ms (Lin et al., 2002). The intensity was progressively increased to elicit the H-reflex (1 mA steps), and then further increased to evoke an M-wave of maximum amplitude. For investigating the homosynaptic post-activation depression (HD), we selected the stimulus intensity eliciting the maximum H-reflex amplitude, as determined by the experiment above (Pierrot-Deseilligny and Burke, 2012). We evoked two trains of 10 consecutive H-reflexes at 0.1 Hz–2 Hz, respectively. Between the two trains (0.1 and 2 Hz) an interval >20 s was respected to avoid the inhibitory effect of the preceding H-responses.

Somatosensory evoked potentials (SEPs) were obtained bilaterally (not simultaneously), by bipolar electrical stimulation of the tibial posterior nerve at ankle. Two sets of 500 stimuli (3 Hz) with an intensity able to induce a slight muscle contraction were delivered. Recordings were performed according to the guidelines and two sets of recordings were obtained for improving accuracy (American Clinical Neurophysiology Society, 2006). The recording electrodes were placed on: ipsilateral popliteal fossa (referred to the patella); T12 s.p. (referred to the iliac crest); C5 s.p. (referred to Fpz’); 1 cm behind Cz on the scalp (referred to Fpz’).

#### Data analysis and statistical analysis

We measured the following variables for each side: M-wave amplitude (mV); M-wave latency (ms); F-wave mean latency (ms); F-wave mean amplitude (μV); H-reflex latency (ms); H-reflex amplitude (μV); Hmax/CMAPmax amplitude ratio; HD at 2 Hz (the average amplitudes of the 2nd–10th H-reflexes, normalized to the average amplitude of the ten H-response evoked at 0.1 Hz); peak-latency (ms) of the popliteal fossa potential (PF), cauda equina potential (N24); N34 potential; P37 potential; interpeak latencies. All values are expressed as mean ± standard deviation (SD).

Each variable was analyzed using a repeated-measures ANOVA with factors: time (T0; T1), modality (sham; active) and side of acquisition (left; right). The threshold for statistical significance was set at 0.05. All calculations were performed in IBM SPSS, version 26.

## Results

### Computational modeling study

The current density and E-field distributions were predicted in the spinal-GM and spinal-WM in the human model considering a current intensity of 4 mA, as applied in the experimental study. The L2 electrode define as was the cathode in all simulations.

**FIGURE 3:**
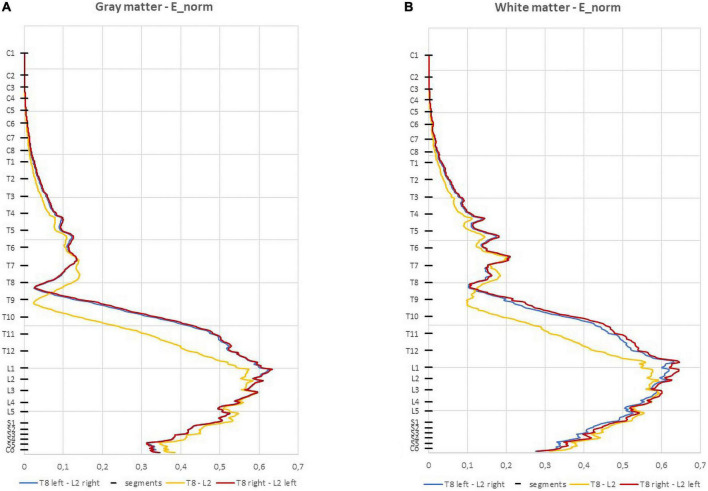
Average electric field (E-field) magnitude in the spinal cord (SC) (V/m) in 1 mm thick transverse slices along the *z* direction in the three montages: **(A)** spinal gray matter (spinal-GM); **(B)** spinal white matter (spinal-WM). The positions of the spinal segments along the SC are indicated in the vertical axis.

The E-field magnitude distribution in the SC is shown in Figure 3. The maximum values predicted in the SC for each montage are:

• T8 left-L2 right—0.634 V/m (GM) and 0.619 V/m (WM);
• T8 right-L2 left —0.634 V/m (GM) and 0.643 V/m (WM);
• T8-L2—0.595 V/m (GM and WM).

In the three montages, maximum E-field is reached in lumbar spinal segments with values above 0.15 V/m for both spinal-GM and WM (Figure 3 and Table 2). The E-field values are also consistent with predictions obtained in previous modeling of experimental studies with observed neuromodulation of cortical and spinal neural responses (Miranda et al., 2013; Fernandes et al., 2019).

**TABLE 2:** Maximum values of the E_norm_ and components E_long_, E_vd_, and E_rl_ and corresponding spinal segments in spinal gray matter (spinal-GM) and WM for the 3 montages (*I* = 4 mA).

Average values	T8 right—L2 left		T8—L2		T8 left—L2 right	
Per 1 mm slice (V/m)	GM	WM	GM	WM	GM	WM
**4 mA**						
E_norm_ | spinal segment	**0.612 | L1**	**0.630 | L1**	**0.571 | L3**	**0.580 | L3**	**0.608 | L1**	**0.609 | L1**
E_long_ | spinal segment	**0.575 | L1**	**0.585 | L1**	**0.534 | L3**	**0.539 | L2**	**0.568 | L1**	**0.578 | L1**
E_vd_ | spinal segment	**0.156 | L1**	0.100 | T6	**0.151 | L3**	0.101 | L3	**0.155 | L1**	0.094 | T6
E_rl_ | spinal segment	0.114 | L1	0.107 | L5	0.115 | L1	0.083 | L5	0.146 | L1	0.048 | L4

Bold values mean statistically significant differences within subjects (*p* < 0.05).

**FIGURE 4:**
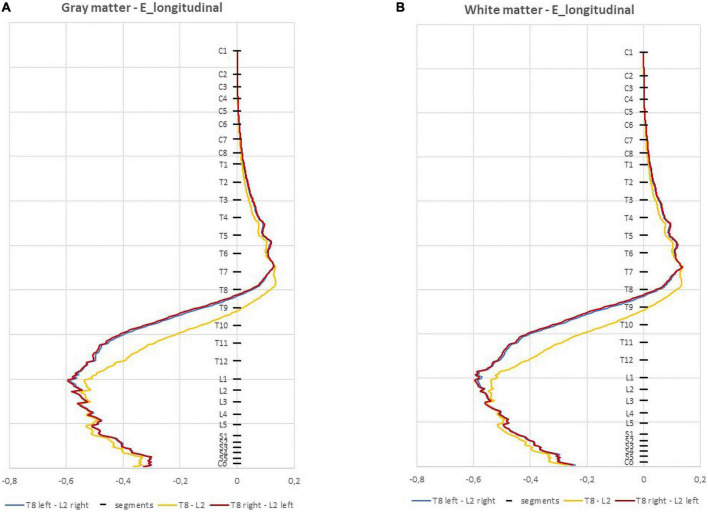
Average electric field (E-field) longitudinal component (V/m) in 1 mm thick transverse slices along the *z* direction in the three montages: **(A)** spinal gray matter (spinal-GM); **(B)** spinal white matter (spinal-WM). Spinal segments positions along the spinal cord (SC) are indicated in the vertical axis.

The paravertebral montages originate higher E-field longitudinal component (E_long_) in both the GM and WM in lower thoracic and upper lumbar regions compared to the spinous montage (L2-T8, see Figure 4; Fernandes et al., 2018). In the sacral region, the spinous montage has slightly higher E_long_ (Figure 4). The maximum values of E_long_ are located on L1 spinal segment in paravertebral montages and on L3 spinal segment in spinous montages (Table 2). E_long_ has almost the same values as the E-field magnitude in each segment, which indicates that the E-field is mostly longitudinally and rostral-caudal oriented from T8 to Co spinal segments between the electrodes (see Figure 4 and Table 2).

The ventral-dorsal component of the E-field (E_vd_) is similar in the GM and WM for the three montages and both are dorsal-ventral oriented in the lumbar region. The maximum values of this component in the spinal WM occur in the lumbar region, however, these are inferior to 0.15 V/m (Table 2).

Regarding the right-left E-field component (E_rl_) the three curves almost overlap in the spinal-GM; the largest differences occur in the spinal-WM. The montage with higher values on E_rl_ in the spinal-GM is T8 left- L2 right with 0.146 V/m in L1 segment. T8—L2 montage has the lowest value, with a maximum value of 0.0834 V/m in the spinal-WM L5 segment (see Table 2). E_rl_ in the spinal-GM shows a decrease in values consistent with shift in L2 position: E_rl_ (L2 left) < E_rl_ (L2 s.p.) < E_rl_ (L2 right). However, maximum values in this component are all below 0.15 V/m in the three montages, which may not be sufficient for a neuromodulatory effect.

**FIGURE 5:**
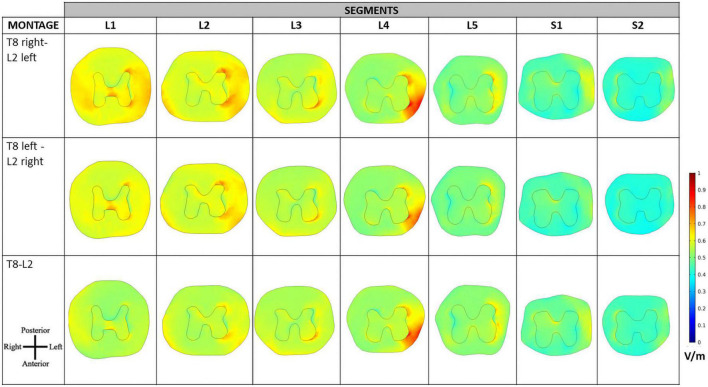
Electric field (E-field) magnitude distribution in transverse slices of the spinal cord (SC). The orientation of the slices is represented in the bottom left corner and color scale in V/m is represented on the right.

The E-field magnitude distribution in transverse slices of spinal segments L1 to S2 is represented in Figure 5 for the 3 montages. Small variations occur in the GM and WM, except for L4 and S1 segments in the WM, where E-field magnitude can vary by 0.2 V/m from left anterior-lateral columns to the remaining regions, especially in T8 right—L2 left montage. T8 right—L2-left and T8-L2 have similar E-field magnitude in spinal-GM and WM, with a left lateralization of the maximum regions in L3 to S2 segments for the 3 montages. These lateral maximum regions correspond to the location of motor descending pathways, specifically the left lateral corticospinal tract and spinocerebellar tracts and in the region of entry and exit of motor and sensitive fibers of the SC (ventral roots and dorsal roots, respectively). The same peak at ventral left region can be justified by a possible CSF narrowing in the spinal canal may be the main anatomical feature causing E-field hotspots.

**FIGURE 6:**
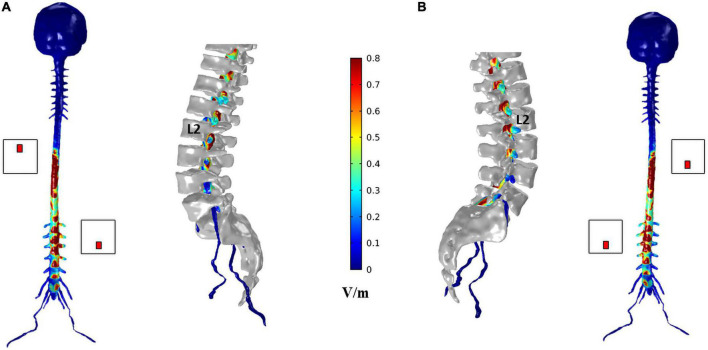
Electric field (E-field) magnitude distribution in the spinal dura and roots for T8 left—L2 right montage **(A)** and T8 right—L2 left montage **(B)**. For each montage, a coronal view of the magnitude on the spinal dura is represented on the left and a sagittal view of spinal dura and vertebra near the L2 electrode position on the right. A color scale for the E-field magnitude is presented in the center.

**FIGURE 7:**
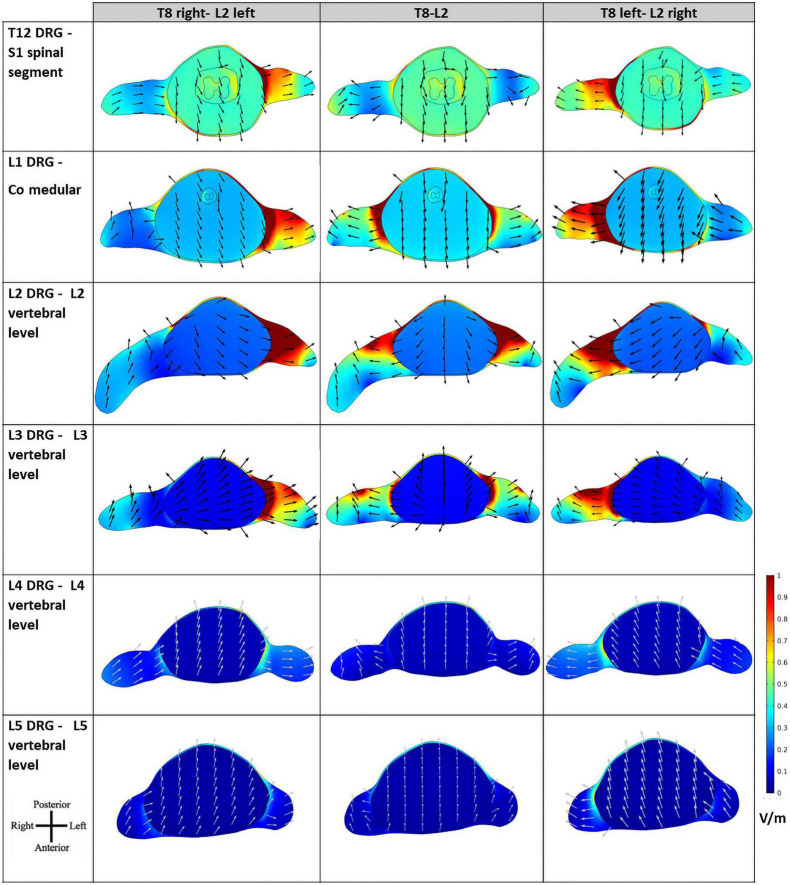
Electric field (E-field) magnitude distribution and direction in transverse slices of the spinal cord (SC) at the levels of the lumbar dorsal ganglia. The orientation of the slices is shown in the bottom left corner and color scale in V/m is represented on the right. E-field direction is indicated by black arrows of the same length.

Despite the similar values observed in transversal slices in paravertebral and spinous montages, the behavior of the E-field differs in the regions close to locations of dorsal roots entry and DRG. The peripheral localization of the DGR compared to the SC can be more favorable to modulation due to current focusing in the intervertebral foramen, resulting in higher E-field exposure (Figure 6). In this case, paravertebral montages may be more efficient than the T8-L2 spinous montage because these originate a more lateralized E-field with larger values around DRG locations. This lateralization is ipsilateral to nearest electrode (L2 in the case of lumbar DGR) with E-field values larger than 1.28 V/m in various ganglia (see Figure 7).

The E-field magnitude distribution in transverse slices of the spinal dura and its interior illustrate the lateralization of the E-field magnitude due to paravertebral montages, at the level of the T12 to L5 DRGs (Figure 7). Maximum values were located in the left and right DRG region in contiguous segments of the sacral region to cauda equina for T8 right- L2 left and T8 left- L2 right montages, respectively (Figure 7). There is no lateralization in the spinous montage (T8-L2). E-field magnitude is higher than 0.15 V/m in the T12 to L3 DRG region for all montages. Only paravertebral montages show values greater than 0.15 V/m in the L4 and L5 DRG regions.

The direction of the E-field at DRG may also be relevant. In T8-L2 montage, the E-field is oriented from the SC to the periphery in both roots; in the paravertebral montages the root ipsilateral to the cathode (electrode place in L2) shows an E-field direction from SC to the periphery, and the contralateral root has an E-field direction from the periphery to the SC (Figure 7). Possibly, there may be some advantage of the T8-L2 montage in stimulating afferent pathways and in paravertebral montages to facilitate the stimulation of efferent pathways or improve communication between hemisections of the spinal cord. For applying in the clinical protocol we selected one paravertebral montage, due to apparent higher afferent pathways excitation.

### Neurophysiological results

Based on the results presented in the previous section we selected the right T8-left L2 montage.

M-waves parameters did not change with time or interventions. Despite the exercise, we observed in both sham and active arms a mild (<1 ms) mean F-wave latency delay at T1 (Pereira et al., 2018, 2022), without any intervention-related change in amplitude.

Somatosensory evoked potentials revealed no change on the right and left sides. Tables 3, 4 summarize these results.

**TABLE 3:** Differences of the neurophysiological responses for lower limbs between T1 and T0 moment (*N* = 14).

Neurophysiological parameters		Mean ± STD—Left		Mean ± STD—Right		Repeated measure ANOVA					
		T1-T0 sham	T1-T0 active	T1-T0 sham	T1-T0 active	*F*	Modality	*F*	Time × modality	*F*	Time × modality × side
M-wave (AH)	Amplitude (mV)	0.19 ± 10.13	0.06 ± 9.78	0.12 ± 10.34	0.18 ± 8.63		n.s		n.s		n.s
	Latency (ms)	0.3 ± 0.71	−0.81 ± 0.59	−1.63 ± 0.63	0.8 ± 0.88		n.s		n.s		n.s
F-wave (AH)	Mean Lat (ms)	0.73 ± 4.59	0.77 ± 4.71	1.07 ± 4.22	1.05 ± 4.28		n.s		n.s		n.s
	Amplitude (μV)	1.07 ± 144.51	−11.02 ± 177.67	−32.07 ± 173.24	−35.23 ± 158.40		n.s		n.s		n.s
H-reflex (soleus)	Lat Hmax (ms)	−0.24 ± 2.27	0.02 ± 2.34	0.01 ± 2.66	0 ± 2.18		n.s		n.s		n.s
	H/M ratio	−0.2 ± 0.31	−0.33 ± 0.29	−0.81 ± 0.28	−1.06 ± 0.25		n.s		n.s		n.s
	Amplitude (μV)	−0.01 ± 4.24	−0.03 ± 5.37	−0.04 ± 4.78	−0.11 ± 3.25		n.s		n.s		n.s
Post-activation depression	0.1 Hz mean (%)	1.41 ± 10.32	4.09 ± 20.86	−0.64 ± 14.61	4.17 ± 21.13	4.782	**0.048^a^**	0.509	0.488	0.059	0.812
	2 Hz mean (%)	−5.37 ± 30.27	−0.02 ± 26.92	−5.6 ± 29.83	0.29 ± 30.81		n.s		n.s		n.s
	0.1 Hz M-wave (%)	5.98 ± 48.94	6.16 ± 54.36	−2.04 ± 46.16	−1.95 ± 51.90		n.s		n.s		n.s
	2 Hz M-wave (%)	2.18 ± 33.01	−3.68 ± 38.07	−6.64 ± 7.73	2.64 ± 9.64		n.s		n.s		n.s
SSEP latency (ms)	PF	0.13 ± 0.94	0.1 ± 1.10	−0.02 ± 1.04	0.17 ± 0.96		n.s		n.s		n.s
	N24	0.04 ± 1.54	−0.08 ± 1.72	0.03 ± 1.60	−0.02 ± 1.72		n.s		n.s		n.s
	N34	0.4 ± 2.84	0.25 ± 2.64	0.19 ± 2.58	−0.01 ± 2.79		n.s		n.s		n.s
	P37	0.22 ± 3.34	−0.13 ± 3.38	−0.16 ± 3.51	0.17 ± 3.47		n.s		n.s		n.s
	PF-N24	−0.1 ± 1.24	−0.18 ± 1.70	0.06 ± 1.19	−0.2 ± 1.60		n.s		n.s		n.s
	N24-N34	0.36 ± 2.00	0.33 ± 2.33	0.16 ± 2.12	0.01 ± 2.23		n.s		n.s		n.s
	PF-P37	0.09 ± 3.21	−0.23 ± 3.38	−0.14 ± 3.38	0 ± 3.23		n.s		n.s		n.s
	N24-P37	0.19 ± 2.43	−0.05 ± 2.70	−0.2 ± 2.71	0.19 ± 2.64		n.s		n.s		n.s

^a^Statistically significant differences within subjects (*p* < 0.05).

Bold values mean statistically significant differences within subjects (*p* < 0.05).

**TABLE 4:** Neurophysiological responses for both lower limb before and after protocol (*N* = 14).

Neurophysiological parameters		Mean ± STD—Left				Mean ± STD—Right			
		T0 sham	T1 sham	T0 active	T1 active	T0 sham	T1 sham	T0 active	T1 active
M-wave (AH)	Amplitude (mV)	17.56 ± 7.3	17.86 ± 7.0	17.94 ± 6.2	17.12 ± 7.6	19.05 ± 7.5	17.42 ± 7.1	17.74 ± 5.1	18.54 ± 6.9
	Latency (ms)	3.09 ± 0.4	3.28 ± 0.6	3.2 ± 0.4	3.26 ± 0.4	3.24 ± 0.4	3.36 ± 0.5	3.21 ± 0.5	3.39 ± 0.7
F-wave	Mean Lat (ms)	47.64 ± 3.0	48.37 ± 3.5	47.6 ± 3.1	48.37 ± 3.6	47.11 ± 2.7	48.18 ± 3.2	47.19 ± 2.8	48.24 ± 3.2
	Amplitude (μV)	263.7 ± 115	264.77 ± 87.5	279.3 ± 117.5	268.28 ± 133.3	301.86 ± 105.8	269.79 ± 137.2	288.31 ± 121.7	253.08 ± 101.4
H-reflex (AH)	Lat Hmax (ms)	28.74 ± 1.6	28.5 ± 1.6	28.46 ± 1.3	28.48 ± 1.9	28.78 ± 2.0	28.79 ± 1.8	28.58 ± 1.6	28.58 ± 1.4
	H/M ratio	0.46 ± 0.2	0.45 ± 0.2	0.5 ± 0.2	0.47 ± 0.2	0.47 ± 0.2	0.43 ± 0.2	0.48 ± 0.2	0.37 ± 0.1
	Amplitude (μV)	5.75 ± 2.9	5.55 ± 3.1	5.56 ± 3.9	5.23 ± 3.6	5.83 ± 3.6	5.02 ± 3.1	5.46 ± 2.5	4.4 ± 2.1
Post-activation depression	0.1 Hz mean (%)	100.45 ± 6.1	101.86 ± 8.3	101.16 ± 15.6	105.25 ± 13.9	96.82 ± 10.9	96.18 ± 9.7	104.09 ± 15.2	108.26 ± 14.7
	2 Hz mean (%)	49.28 ± 18.9	43.91 ± 23.6	43.38 ± 18.4	43.36 ± 19.7	53.2 ± 22.1	47.6 ± 20.1	48.94 ± 24.2	49.23 ± 19.1
	0.1 Hz M-wave (%)	101.9 ± 22.4	107.88 ± 43.5	112.35 ± 34.8	118.51 ± 41.7	106.73 ± 15.5	104.69 ± 48.9	104.91 ± 30.9	102.96 ± 17.8
	2 Hz M-wave (%)	105.58 ± 13.9	107.76 ± 30	105.78 ± 11.4	102.1 ± 36.3	103.48 ± 10.3	96.84 ± 18.1	99.46 ± 9.1	102.1 ± 18.8
SSEP latency (ms)	Pf	8.56 ± 0.6	8.69 ± 0.7	8.55 ± 0.8	8.65 ± 0.8	8.59 ± 0.7	8.57 ± 0.8	8.39 ± 0.7	8.56 ± 0.7
	N24	21.5 ± 1.1	21.54 ± 1.1	21.54 ± 1.2	21.46 ± 1.2	21.54 ± 1.1	21.57 ± 1.2	21.58 ± 1.2	21.56 ± 1.2
	N34	28.06 ± 1.9	28.46 ± 2.1	28.39 ± 1.9	28.64 ± 1.9	28.11 ± 1.7	28.3 ± 1.9	28.49 ± 2.3	28.48 ± 1.7
	P37	38.14 ± 2.4	38.36 ± 2.3	37.95 ± 2.3	37.82 ± 2.4	38.07 ± 2.5	37.91 ± 2.5	37.85 ± 2.6	38.02 ± 2.3
	PF-N24	12.94 ± 0.9	12.84 ± 0.9	12.99 ± 1.3	12.81 ± 1.1	12.94 ± 0.8	13 ± 0.9	13.19 ± 1.1	12.99 ± 1.1
	N24-N34	6.56 ± 1.4	6.92 ± 1.4	6.85 ± 1.7	7.18 ± 1.6	6.57 ± 1.4	6.73 ± 1.6	6.91 ± 1.7	6.92 ± 1.4
	Pf-P37	29.58 ± 2.3	29.67 ± 2.2	29.4 ± 2.4	29.17 ± 2.4	29.48 ± 2.3	29.34 ± 2.4	29.46 ± 2.3	29.46 ± 2.2
	N24-P37	16.64 ± 1.8	16.83 ± 1.7	16.41 ± 2.0	16.36 ± 1.8	16.54 ± 2.0	16.34 ± 1.8	16.27 ± 2.0	16.46 ± 1.7

The amplitude and the latency of maximum H-reflex, H/M ratio did not present a statistically significant difference regarding any intervention modality, side or time (T0/T1; Tables 3, 4).

**FIGURE 8:**
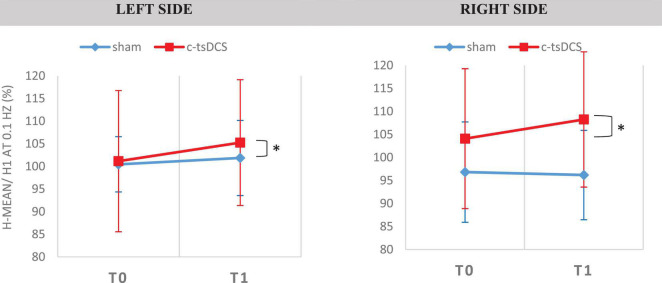
Soleus H-Reflex elicited at 0.1 Hz in the two conditions [Sham and c-tsDCS for the two types of intervention (sham–blue trace and cathodal—red trace)]. H-reflex was obtained in the soleus muscle, for the both sides. [H-mean/H1 at 0.1 Hz (%)—size of mean reflex evoked (%) of mean reflex evoked at 0.1 HZ]. *A statistically significant difference (*p* < 0.05).

We observed a significant increase of the H-reflex amplitude after 0.1 Hz repetitive stimulation between the T0 and T1 after active tsDCS, not dependent on the side [*F*_(1,13)_ = 4.789, *p* = 0.048; Figure 8]. Regarding HD at 2 Hz, we observed no statistical difference in any intervention group (Tables 3, 4).

## Discussion

The present study applied a combined modeling and experimental approach to optimize tsDCS application at lumbar DRG and roots. The DRG can be a relevant modulation target because it also comprises gray matter, constituted by cells bodies of different afferent pathways. Also, axons of motor fibers pass jointly at the intervertebral foramina near DRG location, and focal E-fields may also cause local changes in the membrane potential of these axons. Using lateralized montages in tsDCS can thus provide a novel paradigm for facilitation of spinal pathways by modulating sensory and motor roots at point of entry/exit from the SC.

For that purpose, a computational study was performed in a realistic human model considering one spinous (vertebral) and two paravertebral montages to predict which one maximizes the E-field at the location of lumbar DRG and roots. An experimental study was designed and performed with the montage selected from the modeling study, considering the neurophysiological assessment that enables to evaluate of the excitability of the spinal neurons.

### Computational modeling predictions for the vertebral vs. paravertebral transcutaneous spinal direct current stimulation paradigm

The E-field magnitude predicted for the lumbosacral spinal GM and WM was >0.15 V/m for all montages simulated, which is indicative of a possible neuromodulatory effects, since this value of E-field was predicted for tDCS on the motor cortex in modeling studies reproducing clinical settings with observed changes of motor responses (Nitsche and Paulus, 2000; Miranda et al., 2013). Also, the direction of the longitudinal component was rostral-caudal in all montages from T8 (anode) to Co (cathode) spinal segments (Figure 4), as predicted in previous computational studies (Parazzini et al., 2014; Fernandes et al., 2018; Pereira et al., 2018). The main difference between paravertebral montages and spinous montage was in E-field direction and maxima location rather than in magnitude values. While paravertebral montages originate higher E-field longitudinal components in the lower thoracic and upper lumbar GM and WM, the spinous montage has slightly higher values of this component in the lower lumbar and sacral region (Figure 4 and Table 2). The ventral-dorsal component of the E-field (Evd) is similar in the GM and WM for the three montages and slightly above 0.15 V/m in the lumbar GM (Table 2). The right-left E-field component (Erl) in the three montages had maximum values below 0.15 V/m, which may not be sufficient for a neuromodulatory effect. Erl shows a decrease in values in gray matter consistent with the shift in L2 position: Erl (L2 right) > Erl (L2 s.p.) > Erl (L2 left). The montage with higher Erl values in the spinal-GM is T8 left-L2 right with 0.146 V/m in L1 segment (see Table 2). Differences were also observed in the E-field magnitude in spinal roots and DRG positions, with lateralized maxima in paravertebral montages that do not appear in T8-L2. These lateralized hotspots may be due to the interaction between the large conductivity of CSF and the low conductivity of vertebra (bone), which lead to a high E-field at roots exit from the spinal canal regions located between the electrodes, i.e., in the current’s path. The paravertebral montages may favor the occurrence of these maxima because the placement avoids the low conductive and thick vertebral spinous processes (see Figure 7). Considering the modeling results, we selected T8 right-L2 left montage for our clinical study, to determine experimentally the effects of lateralizing the electric field in tsDCS in functional responses of spinal microcircuits, DRG and roots. The possibility of stimulating the DRG is innovative in the tsDCS paradigm and can be therapeutically relevant, since its efferent axons enter the spinal cord sending collaterals into the gray matter synapsing with IN and projecting caudally and rostrally in white matter (Braz et al., 2014). This oligosynaptic relation may allow to modulate efferent pathways. One current and hypothetic mechanism is through the rectification of hyperexcitability and ectopic firing of primary sensory neurons, more studied in chronic pain (Graham et al., 2022). Lateralization of E-field in paravertebral montages may also differentially facilitate afferent or efferent pathways related to different directions of the E-field in the spinal cord, as disclosed in our model (seen in transversal slices in Figure 7). Previous experimental findings have shown different outcomes when varying electrode polarity and position over the spinal cord (Cogiamanian et al., 2008; Winkler et al., 2010; Lamy et al., 2012; Pereira et al., 2018). tsDCS is a tool that can modulate spinal excitability, depending on the E-field magnitude and orientation relative to spinal neurons. Although tsDCS may lack focality, electrode positioning variations may ensure selective stimulation of longitudinal and transverse pathways of interest, by lateralizing the electrode nearest to the target segment, or both electrodes as demonstrated by our modeling findings.

### Clinical findings

The only significant change was a moderate H-reflex amplitude increase upon 0.1 Hz repetitive stimulation after c-tsDCS (H-reflex facilitation). The M-wave, F-wave, and other H-reflex variables did not show any difference. We speculate that this can derive from depolarizing the resting potential of spinal MNs, increasing spinal MNs activation by exciting afferent Ia fibers, or by improving synaptic efficiency. Previous studies support that c-tsDCS can increase spinal MNs synaptic activation (Ahmed, 2011) and increase their response to stimulation of cutaneous receptors (Alanis, 1952).

However, since other alterations were not disclosed, we cannot reject a chance finding. Indeed, H-reflex modulation by tsDCS still lacks supporting evidence. Some studies with tsDCS showed no change in the HMax/Mmax ratio (Winkler et al., 2010; Cogiamanian et al., 2011; Lamy et al., 2012; Bocci et al., 2015), however, these studies used different montages (thoracic montages, i.e., with the target electrode at T10 or T11 and the return electrode over the right shoulder). Also, tsDCS was not applied simultaneously with exercise. Our subjects were exercised simultaneously to stimulation for enhancing the potential effect of tsDCS, since exercise can reduce interneuron inhibition occurring during immobility (Bussel and Pierrot-Deseilligny, 1977; Taniguchi et al., 2008) and cause descending drive activation (Crone and Nielsen, 1989). Other authors report an increase and a decrease in post-activation depression of the H-reflex (HD), by the application of cathodal and anodal tsDCS for 15 min, respectively (Winkler et al., 2010). These findings were not contraditory to the observed trend in our study, which did not attain significance.

Although in the present study we did not observe any significant difference on SEPs, we cannot exclude a possible influence of tsDCS in the spinal networks and their role in supraspinal activity, which potentiallly interfering with cortical inhibitory networks as showed by Bocci et al. (2014, 2015). The first study in humans using tsDCS was reported in Cogiamanian et al. (2008) applying a T10-right shoulder montage. In this study, the authors reported a reduction of the amplitude of the cervico-medular component (P30) after anodal tsDCS, suggesting that tsDCS can induce changes along human lemniscal pathway conduction. We also performed a previous study on SEP modulation applying cervical tsDCS stimulation, observing a trend to SEPs mean peak latencies delay after anodal tsDCS, with statistical significance for the N9 response (Fernandes et al., 2019).

### Limitations and future studies

The modeling study has some limitations that we should address. The SC is a complex structure with many heterogeneities that can impact on the E-field spatial distribution. To address this issue, we included artificial GM tissue and tensors to represent the anisotropic conductivity of the WM and muscle, based in previous anatomical knowledge of the spinal cord, that lacked in the model used. Future updates of the model should be made based on real segmentation of the spinal-GM and roots and diffusion tensor data to improve the accuracy of morphological and anisotropic spinal characteristics. The inclusion of different tissues that surround the SC, such as fat, CSF, muscle among others (Fernandes et al., 2018) contribute to further reduce possible inaccuracies in the E-field calculations, as pointed out in other modeling studies (e.g., Toshev et al., 2014). Another limitation is that the model does not represent the complex architecture of micro and macrocircuits of the SC, so it does not inform on what happens at cellular level, namely, the interaction between E-field components and spinal neurons and their characteristics. Furthermore, our model does not represent the DRG structures due to the limitations in resolution of the tissue masks used. It only provides an estimate on the intensity and direction of the E-field at DRG location. The difference in scales between macroscale E-field distribution and microscale effects at DRG and cellular levels will require, as future work, the extraction of the E-field vector obtained in our simulations as input parameters of microscale models, following similar methodologies of invasive DRG computational studies, which use models of DRG and neurons with stimulation applied locally (Kent et al., 2018; Graham et al., 2019).

Further modeling studies should address microscale modeling to understand how tsDCS impacts different spinal networks. The value considered as indicative for possible neuromodulation (0.15 V/m) is not a definite value. This value was obtained through macroscale modeling the first cortical DC stimulation montage with observed neuromodulatory effects of the corticospinal pathway (Nitsche and Paulus, 2000; Miranda et al., 2013). More accurate studies should be developed to determine the effective E-field thresholds for neuromodulation regarding different types of neuronal cells and circuits.

Regarding the experimental study, the main limitations were the absence of measurement of amplitude of the SEPs and the small sample size. Measures of amplitude of somatosensory evoked potentials (SSEP) are frequently prone to error since SEPs are very small responses susceptible to contamination by artifacts. The size of our sample is similar to other tsDCS studies, however, we consider this a relevant limitation since a greater number of subjects could define the significance of some of the observed trends.

From our knowledge, this study is the first to evaluate the effects of paravertebral tsDCS on both sensory and motor responses. Future studies using our protocol in patients with UMN dysfunction would be essential to understand its therapeutic potential to address motor-related symptoms such as spasticity.

Intervention protocols guided by computational models can contribute to increase the effectiveness of the tsDCS stimulation over specific spinal target. It is critical to find the best neurophysiological responses that should be used to explore the quality of the results for clinical application. The possibility of influencing the dorsal ganglia function is innovative and can provide a valuable therapeutic solution to address spinal dysfunctions, such as spasticity and chronic pain.

## Data availability statement

The original contributions presented in this study are included in the article/supplementary material, further inquiries can be directed to the corresponding author.

## Ethics statement

The studies involving human participants were reviewed and approved by the Comissão de Ética do Centro Académico de Medicina de Lisboa. The patients/participants provided their written informed consent to participate in this study.

## Author contributions

SF and PM conceived the modeling study. MP and MC conceived the experimental study. SF and MP performed the computational modeling calculations and drafted the manuscript. MP collected all experimental data and analyzed all experimental data. PM and MC offered critical revisions. All authors reviewed, read, and approved the final manuscript.

## Footnotes

1
https://www.materialise.com/en/healthcare/mimics-innovation-suite/mimics

2
www.nlm.nih.gov/research/visible/visible_human.html

3
http://www.fiab.it

4
www.mathworks.com

5
www.comsol.com
